# Gambling in Connecticut adolescents: Prevalence, socio-demographic characteristics, trauma exposure, suicidality, and other risk behaviors

**DOI:** 10.1371/journal.pone.0290589

**Published:** 2025-02-05

**Authors:** Elina A. Stefanovics, Zu Wei Zhai, Marc N. Potenza

**Affiliations:** 1 Department of Psychiatry, Yale University School of Medicine, New Haven, CT, United States of America; 2 U.S. Department of Veterans Affairs New England Mental Illness Research and Education Clinical Center [MIRECC], West Haven, CT, United States of America; 3 National Center on Homelessness Among Veterans, U.S. Department of Veterans Affairs, Tampa, Florida; 4 Program in Neuroscience, Middlebury College, Middlebury, VT, United States of America; 5 Yale Child Study Center, Yale University, New Haven, CT, United States of America; 6 Connecticut Mental Health Center, New Haven, CT, United States of America; 7 Connecticut Council on Problem Gambling, Wethersfield, CT, United States of America; 8 Department of Neuroscience, Yale University, New Haven, CT, United States of America; 9 Wu Tsai Institute, Yale University, New Haven, CT, United States of America; UCL: University College London, UNITED KINGDOM OF GREAT BRITAIN AND NORTHERN IRELAND

## Abstract

Adolescent gambling is a public health concern and has been linked to suicidality, various risk behaviors, and poor health outcomes. However, there is a limited understanding of specific risk and protective factors that may influence gambling behavior in Connecticut adolescents, especially in changing gambling environments. This study examines relationships between adolescents reporting gambling in the past-year and a range of health risk behaviors including vaping, traumatic experiences, academic performance, and receipt of social support. Data from the 2019 Youth Risk Behavior Survey in Connecticut high school students stratified by gambling status were examined in bivariate and multivariate analyses. Among 1,807 adolescents, past-year gambling was reported by 453 individuals [25.4%; 95% confidence interval [CI] = 22.7–28.1%]. Gambling prevalence was higher among older males and lower in adolescents of Asian origin. Gambling was further associated with suicidality and risk behaviors including substance use, smoking [traditional tobacco and electronic vapor use], risky use of digital technologies, unsafe sex, and aggressive behaviors. Gambling was also associated with traumatic experiences, depression/dysphoria, poor academic performance, and less familial social support. The results provide an up-to-date estimate of the current prevalence and correlates of gambling among Connecticut adolescents. The results provide recent estimates of the prevalence and correlates of gambling among Connecticut adolescents. The findings highlight the need for further investigation of specific factors like social support that help with designing and implementing tailored interventions.

## Introduction

Gambling is relatively common activity among adolescents [1]. Despite age restrictions [2], adolescents participate in multiple types of gambling including on lotteries [3], on the internet [4], in video games [5], on electronic-gambling [slot] machines [6], in poker games [7], and in casinos [8]. As gambling becomes more normalized and accessible—and with new gambling opportunities emerging, being heavily advertised and receiving extensive media coverage—adolescents have considerable exposure to gambling. The practice of competitive video gaming, also known as eSports [e.g., using video games as platforms for competition between two or more persons [9], sometimes organized into leagues and tournaments [10], is rapidly becoming popular in public schools across the country [11–13]. Connecticut was the first state to officially welcome eSports into high schools in 2017. High consumption of video games and eSports have been linked to gambling, particularly among male youth [14, 15]. Recent legislative changes regarding the legalization of retail sports and online betting in many U.S. jurisdictions, including Connecticut [16], highlight the need to pay specific attention to gambling among adolescents in current contexts [17]. Some of these changes were preceded by similar gambling-like activities. For instance, before sports gambling was legalized in Connecticut in 2021, daily fantasy sports had been legalized in 2017. Although not legally classified as gambling, the legalization and heavy promotion of daily fantasy sports through various media may have influenced sports-related gambling behaviors, particularly among adolescent males who closely follow sports. Indeed, companies initially involved in daily fantasy sports have now moved into online sports gambling, which was legalized and made available in Connecticut in 2021. After gambling participation by adolescents steadily declined from 2007 to 2017, Connecticut adolescents demonstrated increased gambling participation in 2019 [17].

The growing exposure to and availability of gambling opportunities may pose a significant risk to adolescents, who might not fully understand potential consequences. Although underage gambling can significantly impact adolescents’ lives, it often remains unrecognized by teachers and parents until it reaches problematic levels [18]. Estimates of problem gambling for adolescents have varied. Some studies suggest that between 50% and 70% of adolescents, depending on the country studied, have taken part in some form of gambling at least annually [19], with 0.2%–12.3% of adolescents meeting criteria for problem gambling and 0.2–8.1% among adolescents meeting diagnostic criteria for gambling disorder worldwide [20, 21]. Among Connecticut adolescents, 17.4% were classified as having at-risk gambling and 10.4% as having problem/pathological gambling [22]. Among participants classified as having problem/pathological gambling, 52.3% met full criteria for a gambling disorder [22]. Another survey of Connecticut high school students found that among youth gambling on the internet, 57.5% were classified as having problem/pathological gambling, and among the non-internet-gambling group, 27.7% were classified as having problem/pathological gambling. Almost 11% of Connecticut adolescents acknowledged gambling in casinos in the mid-2000’s [8].

Adolescence is a transitional period from childhood to adulthood marked by biological, cognitive, social, emotional, and behavioral changes [23]. Brain developmental changes during adolescent years have important implications for behavior [24]. Cognitive control is not fully developed and hormonal changes may generate emotional lability and greater responsivities to rewards or stress [25]. Having limited life experience and being more vulnerable to peer influences [26] and marketing campaigns [27, 28], young people may inadequately assess risk levels of activities such as gambling [21, 29]. Changes in adolescents’ lives are also observed in increased social pressure, modifying expectations and needs, formation of new health-related habits, and development of new adaptive skills [30, 31]. Developmental changes during adolescence can link to engagement in problem behaviors [32]. Gambling practices appear to be an early risk behavior, often preceded by experiments with tobacco and illicit drugs or risky sex practices [21], as adolescents may consider gambling less risky than alcohol or cigarettes, and more available than alcohol or marijuana [33]. Adolescence is a time when many youths learn how to regulate their emotions and develop personal coping skills for dealing with stress. Some adolescents may deal with stress by engaging in risky behaviors. In such cases, gambling may appear as an attractive means to escape from stressors.

According to the problem behavior theory [PBT] that describes behavioral patterns in adolescents [34], risky behaviors, including gambling, result from person-environmental interactions and often co-occur [35], where both internal and external [e.g., environmental] factors may contribute to the engagement in problematic behaviors [36], and where the balance between risk and protective factors determines whether or not the individual will engage in problematic behaviors. In contrast to theories focusing on how engagement in one risk behavior may displace other activities, the PBT suggests that as one type of problematic behavior increases, the likelihood of the occurrence of other problem behaviors also increases. Co-existence of these behaviors may also be explained by common underlying risk factors, supporting the notion of a problem behavior syndrome [32, 37]. Sociodemographic characteristics of adolescents who gamble include older age and male gender, with older adolescent boys tending to gamble more often and bet higher amounts of money [38].

Although the PBT suggests that one risk behavior may elevate the likelihood of others, most studies have not compared a full range of potentially co-occurring problem behaviors in the same analysis, particularly as new emerging risk behaviors related to digital technology use and electronic delivery of drugs have become more prevalent. The present investigation seeks to address gaps in the existing research and uses recently collected data to examine the associations between past-year gambling and other health risk behaviors, including substance use, tobacco and electronic vapor product use, sexual behaviors, risky use of digital technologies, and risk activities on school property. To do so, we interrogated data from the year prior to the COVID-19 pandemic. Specifically, in the current study, we sought to provide insight into adolescent gambling and risk behaviors in a contemporary, representative sample of Connecticut high-school students participating in the 2019 Youth Risk Behavior Survey [YRBS].

In this exploratory study, we had two specific aims: [1] provide an up-to-date, pre-pandemic estimate of the prevalence of past-year gambling among Connecticut high-school students; and [2] identify sociodemographic characteristics associated with gambling and assess trauma exposure, other risk behaviors, suicidality, homelessness, health status, academics, and social support associated with gambling. Based on the PBT, we hypothesized that adolescents who reported past-year gambling would have low academic grades and be more likely to engage in problem behaviors, including alcohol and drug use, tobacco, vaping, risky sex practices, and aggressive behaviors [e.g., physical fights and carrying weapons on school territory]. We hypothesized that adolescents who experienced low levels of parental support would be more likely to engage in problem behaviors as more social support may help develop better coping mechanisms and emotional regulation skills. Finally, we hypothesized that gambling participation would be prevalent particularly among older adolescent males and associate with trauma exposure and suicidality.

## Materials and methods

### Participants

Cross-sectional YRBS data collected in 2019 from public high-school students in Connecticut were analyzed. The YRBS is a biennial, cross-sectional, school-based survey. The YRBS is part of the Youth Risk Behavior Surveillance System, developed by the Centers for Disease Control and Prevention [CDC] to monitor health behaviors in six key domains: injuries and violence, sexual behavior [related to unintended pregnancy and sexually transmitted diseases], tobacco use, alcohol and other drug use, unhealthy dietary behaviors, and inadequate physical activity [http://www.cdc.gov/yrbss]. Altogether, 2,015 students from 33 public, charter, and vocational high schools in Connecticut were surveyed during the spring of 2019. Students completed a self-administered, anonymous, 99-item questionnaire collecting data about demographics characteristics and participation in risk behaviors including ones potentially contributing to unintentional injuries and violence, sexual behaviors potentially contributing to unintended pregnancies and sexually transmitted diseases, alcohol, tobacco and other drug use, and poor physical activity. A description of data collection procedures in the YRBS can be obtained elsewhere [http://www.cdc.gov/yrbss].

The survey procedures have been previously described [39]. Briefly, the school response rate was 66%, the student response rate was 82%, and the overall response rate was 54%. The results were representative of all students in grades 9–12. Permission for the survey was obtained through school administrations. Initially, school superintendents were notified regarding selection of their schools to participate. Following superintendent approval, permission from school principals was obtained. Before survey administration, parental permission was obtained. Teachers and/or students of selected classrooms could decline participation. Parents were mailed letters outlining the study, and letters directed parents to contact their schools should they wish to decline their child’s/children’s participation. Active consent, in which parents affirmed and authorized permission, was obtained when required. All procedures were performed in accordance with the 1964 Helsinki Declaration and its amendments. Surveys were administered at each school in a single day. Answers were anonymous and confidential, and students were reminded that participation was voluntary. No personal identifying information was collected. School and classroom codes were removed from the final dataset. The Center for Disease Control [CDC] cleaned and coded the dichotomous variables from the raw data in the final data set. Survey procedures were designed to protect privacy of the students by allowing for anonymous and voluntary participation. During survey administration, students completed the self-administered questionnaire during one class period and recorded their responses directly on a computer-scannable booklet. The CDC’s Institutional Review Board approved the protocol for the YRBS. This research has been deemed exempt from Yale University School of Medicine IRB review by the Human Subjects Committee. This protocol has been determined to be exempt under federal regulation 45 CFR 46.101[b] [2]. Exempt studies do not require annual IRB review.

### Measures

***Socio-demographics characteristics*** included age (≤15 years, 16–17 years, ≥18 years), sex (female/male), and race/ethnicity (Black, Hispanic/Latino, White, and Other).

***Gambling*** was assessed with the question, “During the past 12 months, how many times have you gambled on a sports team, gambled when playing cards or a dice game, played one of your state’s lottery games, gambled on the Internet, or bet on a game of personal skill such as pool or a video game?” If respondents gambled one or more times during the past 12 months, they were classified as having gambled; otherwise, they were classified as not having gambled.

***Trauma variables*:** Adolescents were assessed on trauma-related measures including feeling unsafe at school in the past 30 days, as well as having experienced threat or injury with a weapon at school, physical fighting, dating violence (sexual and/or physical assault by a dating partner), bullying at school, and electronic bullying (bullied through texting and social media) in the past 12 months; and ever having been forced into sexual intercourse in one’s lifetime. Homelessness was measured by the question, “During the past 30 days, did you ever sleep away from your parents or guardians because you were kicked out, ran away, or were abandoned?”

***Suicidality*:** Adolescents were assessed on past-year suicide attempts [yes/no] and suicidal ideation (yes/no).

***Risky use of digital technologies*** was assessed using 3 questions. Adolescents were asked a questions concerning ***video game and computer use*** other than for school purposes for more than 3 hours daily (yes/no) and whether they were ***talking on a cell phone or texting/e-mailing*** while driving in the past 30 days (yes/no).

**Substance use. *Medication misuse*** included questions about ever having taken over-the-counter medications to get high (yes/no).

***Binge drinking*** was assessed as heavy drinking of alcohol [≥ 4 drinks in a row for females, ≥ 5 drinks in a row for males] one or more times in the past 30 days [yes/no].

***Lifetime actual and synthetic marijuana use*** was assessed with the questions, “During your lifetime, how many times did you use marijuana [synthetic marijuana]?” Responses were coded as 0 in cases of “never” and 1 if other options were chosen.

***Lifetime electronic vapor use*** was dichotomized using the question, “During your lifetime, how many times did you use electronic vapor?” Responses were coded as 0 in case “never” and 1 if other options were chosen.

***Current marijuana and alcohol use*** was assessed using single survey items [“During the past 30 days, how many times did you use marijuana/alcohol?”] and coded dichotomously yes/no, with a “no” response defined as a “never” response.

**Tobacco and electronic vapor use. *Current tobacco use*:** Two questions concerned the use of any cigarettes or cigars in the past 30 days (yes/no).

***Current electronic vapor use*** was assessed with the questions, “During the past 30 days, did you use any electronic vapor product?” and “use them on the school property?” (yes/no).

***Risky sexual behavior*** was assessed with two questions: “Have you ever had intercourse with four or more persons?” and “Did you drink alcohol before the last time you had sex?” (yes/no).

**Aggressive behavior. *Risk activities on school property*** were assessed with two questions assessing having carried a gun, knife, or club (yes/no) or been offered drugs at school (yes/no) during the past 30 days.

***Physical fights*** reflected past-12-month involvement in a physical fight (yes/no).

***Health-related variables*.** Questions included a student’s subjective consideration of general health as good (yes/no) and sleeping 8 hours or more daily (yes/no). Depressive symptoms were assessed with the question, “In the last 12 months, did you feel sad or hopeless almost every day for more than 2 weeks?”

***Academics*** were assessed with two questions investigating involvement in special education programs (yes/no) and grade average (mostly grades of A or B vs. lower).

***Social support variables*** included family support measured by the question, “Do you agree that your family loves you and gives you help and support when you need it?” Teacher support was measured by the question, “Is there at least one teacher or other adult at school that you can talk if you have a concern?”

### Data analysis

Analyses proceeded in three steps. First, to summarize variables, descriptive statistics were computed. Sociodemographic variables that differed by group in bi-variate analyses at p < .01 were adjusted in subsequent multivariable analyses. Second, Bonferroni-corrected χ^2^ and analyses of variance (ANOVAs) were conducted to compare characteristics (e.g., substance use, tobacco use. electronic vaping, risky sexual behavior, risky use of digital technologies, aggressive behaviors, suicidality/self-injury, trauma variables, subjective health status, academics, and social support) between groups (gambling vs. non-gambling). Third, unadjusted and adjusted weighted frequencies of variables were calculated. Fourth, a series of multivariable binary logistic regression analyses were conducted to assess relationships and independent associations between group membership and substance use, risky behavior, suicidality/self-injury, trauma variables, health status, academics, and social support measures. These analyses were adjusted for sociodemographic variables (age, sex, and race/ethnicity). All analyses were performed using SAS 9.4 statistical software (SAS Institute Inc., Cary, North Carolina, U.S.) [40]. Statistical significance for comparisons of sociodemographic measures was set at *p* < .01 (2-tailed), and for subsequent multivariable analyses significance was set at p = 0.001 (α_adjusted_ = α/n; where α is the original α level [0.05) and n is the total number of comparisons; 0.05/33 = 0.001). To make statistically valid inferences from the sample to the study population, we analyzed data considering the sample design and sample-calculated weights. The survey procedures were employed to compute variances that accurately reflected the complex sample design and estimation procedures. Consequently, sample proportions were based on weighted percentages.

### Ethics

The high-school survey and procedures were approved by the Yale School of Medicine IRB, and all procedures were approved by the participating high-schools. Passive consent procedures were adopted for parental consent. Parents of students were notified by mail of the survey and that to exclude their child’s participation in the study, they should contact the school or the study team. Parental permission for their child’s participation was implied if they did not make contact with the team or school. Students were informed at the time of survey administration that it was being used for a study, that their participation was fully voluntary, and that they could refuse to fill out the survey if they wished. Those who did not participate in the survey were allowed to do schoolwork while others worked on the survey. Students were also told not to include identifying information on the survey to maintain anonymity. Students were given a pen to complete the survey. Procedures were in accordance with the Declaration of Helsinki 2013.

## Results

### Descriptive statistics

Data were available for 2,015 individuals. Respondents who had missing data on the gambling question (n = 208; 10%) were excluded from analyses. Thus, the total analytic sample consisted of 1,807 individuals, of whom 891 (49.7%) were males. Approximately one-quarter [n = 453; 25.4%] reported gambling one or more times during the past 12 months. Slightly more than one-third of the sample (n = 733, 34.3%) was 15 years old or younger. Almost half of the sample included 16- and 17-year-old students (n = 855; 45.7%), with the remainder of the sample (n = 213; 11.8%) being aged 18 years and older. Slightly more than half of the sample (n = 902; 57.7%) was White; 11.6% (n = 159) was Black; 5.1% (n = 121) was Hispanic; 3.7% (n = 84) was Asian; and 21.9% was multiracial or other race (n = 525).

**Fig 1:**
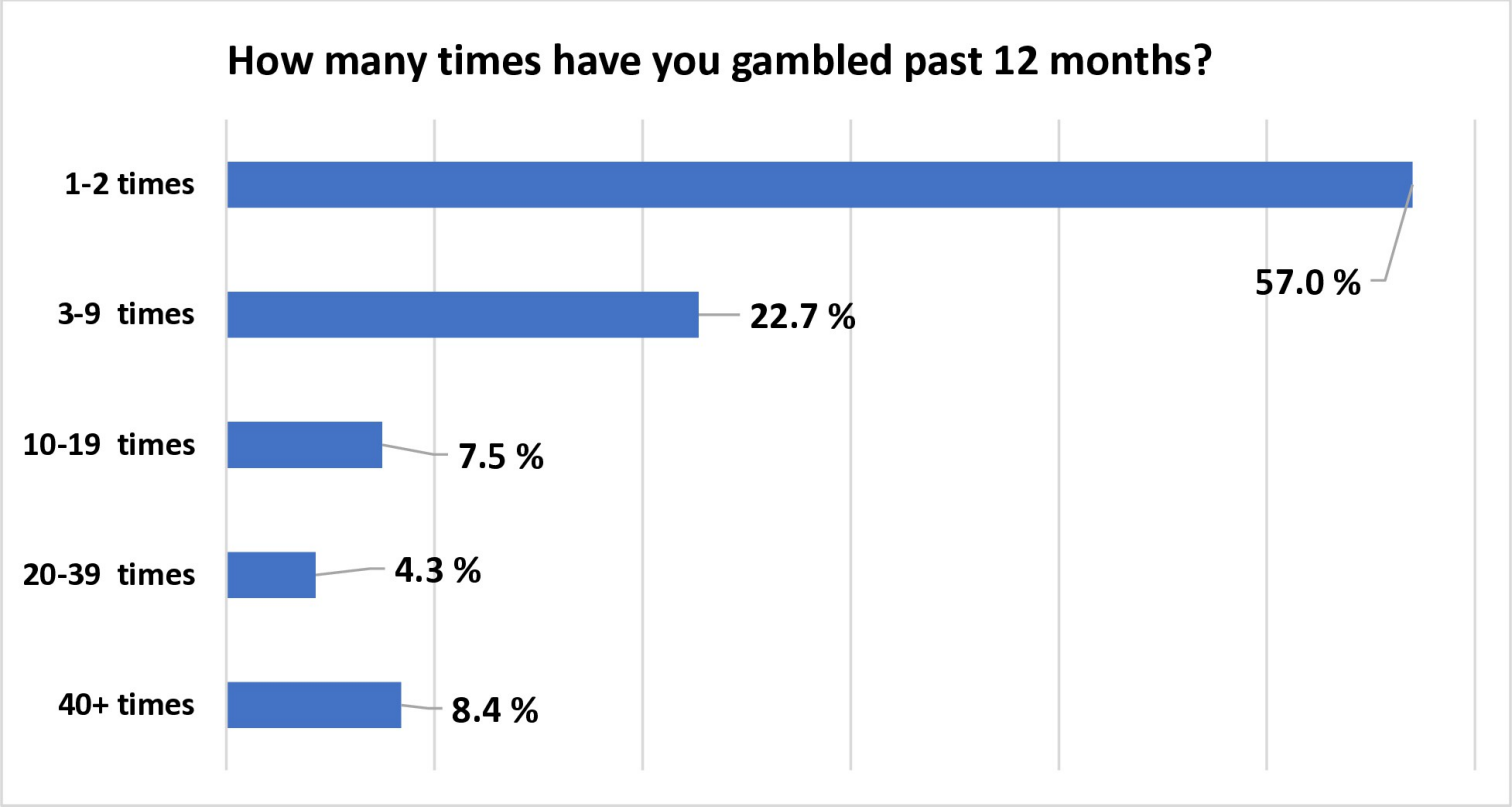
Self-reported gambling frequency among adolescents over the past 12 months. This bar graph shows the self-reported number of times adolescents have gambled over the past 12 months. The majority (57%) reported gambling 1–2 times, followed by 22.7% gambling 3–9 times. Smaller percentages engaged in gambling 10–19 times (7.5%), 20–39 times (4.3%), and 40+ times (8.4%).

The gambling patterns among persons who reported having gambled in the past 12 months are presented in *Fig 1, Table 1*.

**Table 1:** Self-reported gambling frequency among adolescents over the past 12 months.

Gambling Frequency	Percentage of Adolescents (%)
1–2 times	57.0
3–9 times	22.7
10–19 times	7.5
20–39 times	4.3
40+ times	8.4

**Bivariate and multivariate analyses.** *Table 2* displays the sociodemographic characteristics of adolescents with and without gambling. Adolescents who reported past-year gambling were more likely to be older and male.

**Table 2:** Socio-demographic characteristics of the sample stratified by gambling status [N = 1,807].

	Reported past-year gambling		Did not report past-year gambling		Statistical Test		Reported past-year gambling vs. did not report past-year gambling		
Variable	n = 453; 25.4%*		n = 1,354; 74.6%*				OR	95% Confidence Limits	
	n	%*	n	%*	Rao-Scott χ2	p			
***Age in Years***					22.91	< .0001			
15 and younger	167	34.86	566	40.02			ref		
16–17	200	46.37	655	50.52			1.05	0.82	1.36
18 and older	84	18.77	129	9.46			**2.28**	**1.47**	**3.52**
***Sex***					75.92	< .0001			
Male	303	67.81	588	43.51			**2.74**	**2.09**	**3.57**
Female	147	32.19	761	56.49			ref		
***Race/ethnicity***					9.58	0.04			
White	220	56.02	682	58.31			ref		
Black	49	13.91	110	10.85			1.33	0.81	2.19
Hispanic	30	4.92	91	5.12			1.00	0.55	1.82
Asian	11	1.67	73	4.34			0.40	0.22	0.75
Multiracial and other	141	23.48	384	21.37			1.14	0.82	1.59

*Weighted percent; significant findings are bolded

*Table 3* presents suicidality, traumatic experiences, substance use, and other risk behaviors stratified by gambling status and adjusted for age and sex [S1 Table in S1 File for unadjusted findings].

**Table 3:** Traumatic experiences, suicidal and other risk behaviors stratified by gambling status [N = 1,807].

	Reported past-year gambling		Did not report past-year gambling				Reported past-year gambling vs. did not report past-year gambling		
Variables	n = 453; 25.4%*		n = 1,354; 74.6%*		Statistical Test				
	n	%*	n	%*	Rao-Scott χ2	p	AOR**	95% Confidence Limits	
***Traumatic experiences***									
Bullied at school	94	19.54	236	17.80	0.72	0.40	1.31	0.97	1.77
Electronically bullied	95	20.55	171	12.55	14.27	0.0002	**2.15**	**1.61**	**2.89**
Homelessness	68	15.48	60	3.97	93.40	< .0001	**4.74**	**3.15**	**7.13**
Experienced forced sex	49	10.79	64	4.40	30.26	< .0001	**3.67**	**2.51**	**5.36**
Threatened or injured with weapon at school	48	10.61	78	5.38	8.36	0.01	2.09	1.21	3.59
Felt unsafe at school	54	11.15	78	5.55	22.56	0.002	2.22	1.41	3.50
Experienced physical or sexual dating violence	69	21.39	109	14.02	5.65	0.017	2.18	1.37	3.46
***Substance use***									
Buy over-the-counter medications to get high	46	10.18	37	2.47	38.60	< .0001	**4.07**	**2.55**	**6.49**
Binge drinking [PM]	105	24.27	124	9.42	53.67	< .0001	**3.30**	**2.49**	**4.37**
Alcohol use [PM]	168	38.81	279	21.59	38.72	< .0001	**2.53**	**1.89**	**3.39**
Marijuana use [PM]	135	30.99	250	18.70	13.72	0.0002	**1.98**	**1.36**	**2.89**
Marijuana use [LT]	207	48.82	423	31.85	34.73	< .0001	**2.26**	**1.74**	**2.93**
Synthetic marijuana use [LT]	55	12.70	53	3.96	32.74	< .0001	**3.85**	**2.24**	**6.61**
***Tobacco and electronic vapor use***									
Cigarettes use [PM]	34	7.56	29	2.21	23.49	< .0001	**3.52**	**1.87**	**6.62**
Cigar use [PM]	47	10.75	18	1.39	105.51	< .0001	**6.25**	**3.25**	**12.02**
Electronic vapor product use [PM]	155	38.24	305	23.69	25.87	< .0001	**2.19**	**1.65**	**2.93**
Electronic vapor product use [LT]	253	57.79	540	40.36	36.81	< .0001	**2.21**	**1.72**	**2.82**
Electronic vapor use at school [PM]	93	20.29	110	8.20	36.14	< .0001	**2.92**	**2.07**	**4.12**
***Risky Sexual behavior***									
Multiple sex partners	46	11.91	70	5.66	15.70	< .0001	**1.87**	**1.16**	**3.02**
Having sex while drunk	41	31.05	49	16.75	12.11	0.0005	**2.59**	**1.63**	**4.09**
***Risky use of digital technologies***									
Play video games or use computer not for school	402	89.43	1086	80.08	12.65	0.0004	**1.73**	**1.12**	**2.68**
Talking on cell phone while driving	119	51.97	181	31.28	58.53	< .0001	**2.31**	**1.77**	**3.01**
Texting/e-mailing while driving	96	45.92	143	26.79	26.79	0.0002	**2.15**	**1.53**	**3.02**
***Suicidality***									
Attempted suicide	50	10.77	71	5.14	20.11	< .0001	**2.82**	**1.87**	**4.26**
Considered suicide	72	15.76	165	11.76	3.87	0.049	1.65	1.12	2.43
***Aggressive behaviors [PM]***									
Risk activity on school property [carried a weapon on school property	31	6.61	31	2.22	31.21	< .0001	**2.63**	**1.66**	**4.15**
Physical fight	155	34.65	201	14.19	105.25	< .0001	**3.09**	**2.32**	**4.11**

*weighted %

** AOR adjusted for age and sex; significant findings according to Bonferroni correction are bolded

PM = past month

LT = lifetim

Adolescents who reported past-year gambling experienced more trauma, including electronic bullying, forced sex, having felt unsafe at school, and homelessness. The gambling group was more likely to report alcohol and binge drinking, marijuana and synthetic marijuana use, and use of over-the-counter medication to get high. The gambling group was more likely to report having played video games or using a computer for other than school purposes three or more hours per day, using alcohol and/or marijuana (current and lifetime), engaging in binge drinking, and misusing over-the-counter medication to get high. They were also more likely to report having used tobacco (cigarettes and cigars) and electronic vapor products, been offered drugs at school, engaged in risky sexual behaviors (sex with multiple partners and sex while drunk), and involvement in aggressive behaviors (e.g., carrying a weapon on school property, having been in a physical fight on school property). Adolescents from the gambling group reported they were more than twice as likely to have attempted suicide.

*Table 4* presents subjective health, academic performance, and social support measures adjusted for age and sex [S2 Table S1 File for unadjusted findings].

**Table 4:** Perceived health status, academics and support variables stratified by gambling status [N = 1,807].

	Reported past-year gambling		Did not report past-year gambling					Reported past-year gambling vs. did not report past-year gambling	
Variables	n = 453; 25.4%*		n = 1,354; 74.6%*		Statistical Test				
	n	%*	n	%*	Rao-Scott χ2	p	AOR**	95% Confidence Limits	
***Perceived health status***									
Dysphoria/depression	148	31.53	428	30.50	0.18	0.04	1.34	1.01	1.77
Considered general health as good	265	61.15	738	55.57	5.01	0.17	1.17	0.93	1.47
Slept 8 hour or more	85	18.78	293	21.79	1.30	0.21	0.80	0.55	1.16
***Academics***									
Mostly A and B grades	308	68.68	1105	82.82	42.47	< .0001	**0.50**	**0.36**	**0.69**
Received special education	51	10.16	153	11.30	0.33	0.56	0.92	0.57	1.49
***Social Support***									
Family support	357	79.80	1165	86.85	16.00	< .0001	**0.58**	**0.41**	**0.82**
Teacher support	265	58.28	881	65.18	6.21	0.01	0.73	0.58	0.92

*Weighted

**AOR adjusted for age and sex; significant findings according to Bonferroni correction are bolded

The gambling group reported poorer school performance and less family support.

## Discussion

This study examined 2019 data regarding gambling behaviors and their correlates among high-school students in Connecticut. Approximately one-quarter [25.4%] reported past-year gambling. This estimate appears higher than that previously found [18.6%] among Connecticut adolescents surveyed in 2017 [39]. These differences may reflect, in part, the use of slightly different wording in the question to assess gambling involvement in the past year in these two samples. However, other possibilities [e.g., the increasing popularity and social acceptability of sports gambling, especially following the introduction of daily fantasy sports in Connecticut in 2017 and their promotion through frequent advertisements] warrant consideration. The observed gambling prevalence estimate is lower than estimates based on national telephone-based surveys [44.3% to 68%] and in the low range of what was has been reported among students in school-based surveys [24.4% to 86%] and convenience samples [22.5% to 47.4%], although changes over time in the prevalence of adolescent gambling during the early part of the second decade of this millennium should also be considered [41, 42]. While half of the adolescents who gambled last year reported doing it only 1 or 2 times, 12.7% of gambling adolescents reported gambling 20 or more times, with 8.4% reporting more than 40 times.

Past-year gambling was higher among male adolescents, consistent with previous findings [20, 43, 44]. Boys generally gamble more and are more likely to experience gambling problems compared to girls [45]. Additionally, men with gambling problems often report beginning their gambling activities in their teens, while women typically start gambling later in life [46]. Understanding gender-related differences can inform effective educational strategies for families and school administrators and counselors, helping them to shape preventive efforts with sex-informed considerations. In support of our hypothesis, and consistent with the PBT, adolescents who gambled in the past year reported more frequent engagement in other problem behaviors including alcohol, tobacco, and marijuana use, similar to prior observations [22, 41, 47]. Such gambling/substance-use relationships were found to extend to use of electronic vaping devices, a newer substance-related concern among youth [48]. Further, gambling in adolescents was associated with other risk behaviors including unsafe driving (using cell phone/e-mailing/ texting while driving) and frequent use of computers or cell phones and risky sexual behavior. Gambling was also associated with aggressive behaviors including illegal activities on school grounds and getting into physical fights, as in prior studies of Connecticut high-school students [22]. The association between problem-gambling severity and weapon-carrying is also consistent with previous studies of Connecticut high-school students [22, 39].

Consistent with some prior findings, adolescents reporting past-year gambling, relative to those reporting no gambling, were more likely to report lifetime suicide attempts. One recent study associated gambling and suicide attempts, with the likelihood of suicide attempts increasing with increasing problem-gambling severity in both young women and men [49]. A history of suicide attempts was found to be independently associated with multiple risk-taking behaviors such as gambling, alcohol, nicotine and other drug use, and aggressive behavior [50]. Other studies have also associated health risk behaviors with suicidal ideation and attempts among adolescents, and recognition of these health risk behaviors may be one means of identifying those who may be at increased risk of suicidality [51].

Traumatic experiences were more prevalent in the gambling group and included bullying, physical violence, forced sex, and homelessness. These findings resonate with earlier findings involving adolescents [1]. Youth with gambling problems typically report having experienced more negative life events relative to those who do not gamble [52–54]. Adolescents with past-year gambling also reported low social support. Specifically, they have reported less family support and love from their parents. Social support may influence relationships between gambling and other risk-taking behaviors [55]. Specifically, social support from parents, teachers, and friends may decrease the likelihood of involvement in risky behaviors including gambling [54–58]. Supportive social environments may promote emotional well-being, buffer stress [59], and promote coping, mitigating associations between stress and anxiety [60]. Social support may also contribute to higher self-esteem and resilience [61], while low social support has been associated with greater problem-gambling severity, mental health problems and perceived stress [62].

Several strengths and limitations of the study are noteworthy. The YRBS is a large epidemiological study involving self-report measures. Self-reported data are subject to biases, such as reliability on memory, social desirability, and honesty of responses. Given the illegal nature of several variables (e.g., gambling among participants younger than 18 years, illicit substance use), it is also possible that participants may have not reported engagement in certain behaviors, leading to overly conservative prevalence estimates. Alternatively, over-reporting of risk behaviors (e.g., for bravado]) may also have occurred. We have classified gambling groups based on the single question, “During the past 12 months, how many times have you gambled on a sports team, gambled when playing cards or a dice game, played one of your state’s lottery games, gambled on the Internet, or bet on a game of personal skill such as pool or a video game?” This classification does not allow for identifying gambling groups based on types of gambling or problem-gambling severity, or for assessing related impacts. Additional studies using more dynamic classifications are needed. Second, the cross-sectional design precludes analyses or statements about potential directionality or causality. Third, although this is a representative sample, the survey was conducted in select school districts in one state, and the extent to which our findings generalize to populations outside the state of Connecticut is unclear. Fourth, many questions assessing non-gambling domains were not validated scales. While this may have reduced subject burden, additional studies using empirically validated measures are needed. Fifth, the categories of variables could have been defined differently (for example, suicide attempts may have been considered as traumatic, but was grouped with other suicidality measures). Sixth, the consent procedures used may influenced findings. Seventh, COVID-19 pandemic and related stress and combative measures (spatial distancing, closures of schools) may have influenced gambling, other risk behaviors and mental health [63, 64]. Thus, additional studies during and following the pandemic are needed.

## Conclusion

Adolescents who have engaged in past-year gambling often participate in other risk-taking behaviors, including newer ones that include electronic delivery of substances and use of digital technologies. Links between risk-taking behaviors and gambling increase evidence from earlier research showing associations between sexual risk-taking [65], substance use and violence [39, 66], binge drinking, and gambling [67]. An association between gambling and health risk-taking also supports the notion that participation in one risky behavior may increase participation in others, consistent with the PBT model [68]. Given that some of these behaviors occur on school grounds, school administrators and teachers should be aware of them and their implications for adolescent health [69]. Early life stressors, adverse childhood experiences and trauma warrant screening and assessment. Adequate social support may function as a protective factor against gambling and problem gambling as it enhances healthy coping mechanisms. Hence, strengthening adolescents’ social support from school personnel and parents may be beneficial. Social support may be a crucial component of intervention strategies for adolescent gambling and its correlates. Strong familial relationships and open communication between adolescents and parents, may reduce the likelihood of involvement in risky behaviors overall and in gambling in particular. Adolescent gambling should be considered an important public health issue because it clusters with other unhealthy behaviors, which may increase the risk of negative health outcomes over time. Prevention and intervention efforts should not solely focus on gambling but should also take into consideration other simultaneous risk-taking behaviors. Prevention strategies that aim to increase social support, especially from parents, may be helpful in reducing adolescent gambling and related concerns.

Overall, results suggest that gambling is a relatively common activity among adolescents. Given that adolescents may be increasingly exposed to various gambling opportunities, especially online, it is important to identify individuals at risk, monitor the consequences of gambling, and refer individuals for help when needed. This may be particularly relevant in Connecticut and other states in which online gambling and sports gambling have been recently legalized.

## Supporting information

S1 File *Table 1*. Traumatic experiences, suicidal and other risk behaviors stratified by gambling status [N = 1,807] *and Table 2*. Perceived health status, academics and social support stratified by gambling status [N = 1,807].
(DOCX)
