# Supplementary material for: Gambling in Connecticut adolescents: Prevalence, socio-demographic characteristics, trauma exposure, suicidality, and other risk behaviors
Source: PLoS One. 2025 Feb 5;20(2):e0290589. doi: 10.1371/journal.pone.0290589 (PMC11798454; doi:10.1371/journal.pone.0290589)
Supplement: S1 File — Table 1. Traumatic experiences, suicidal and other risk behaviors stratified by gambling status [N = 1,807] and Table 2. Perceived health status, academics and social support stratified by gambling status [N = 1,807]. (DOCX) [file pone.0290589.s001.docx]

| **Supplemental Table 1. Traumatic experiences, suicidal and other risk behaviors stratified by gambling**  **status [N=1,807]** | | | | | | | | |  |
| --- | --- | --- | --- | --- | --- | --- | --- | --- | --- |
|  |  | **Reported past-year gambling vs.**  **did not report past-year gambling** | | | | |  |  |  |
| **Variables** | **Statistical Test** | | | | |  | |  |  |
|  | **Crude Odds Ratios** | | **95% Confidence Limits** | | | | |  |  |
| ***Traumatic experiences*** |  | | |  |  | | |  |  |
| Bullied at school | 1.12 | | | 0.84 | 1.49 | | |  |  |
| Electronically bullied | **1.80** | | | **1.34** | **2.42** | | |  |  |
| Homelessness | **3.79** | | | **2.39** | **6.02** | | |  |  |
| Experienced forced sex | **2.63** | | | **1.72** | **3.97** | | |  |  |
| Threatened or injured with weapon at school | **2.08** | | | **1.40** | **3.10** | | |  |  |
| Felt unsafe at school | **2.14** | | | **1.45** | **3.15** | | |  |  |
| Experienced physical or sexual dating violence | 1.61 | | | 1.07 | 2.42 | | |  |  |
| ***Substance use*** |  | | |  |  | | |  |  |
| Buy over-the-counter medications to get high | **4.48** | | | **2.29** | **7.18** | | |  |  |
| Binge drinking [PM] | **3.08** | | | **2.27** | **4.18** | | |  |  |
| Alcohol use [PM]  Marijuana use [PM]  Marijuana use [LT]  Synthetic marijuana use [LT] | **2.03**  **1.95**  **2.04**  **3.53** | | | **1.79**  **1.50**  **1.61**  **2.32** | **2.95**  **2.53**  **2.58**  **5.37** | | |  |  |
| ***Tobacco and electronic vapor use*** |  | | |  |  | | |  |  |
| Cigarettes use [PM] | **3.64** | | | **1.07** | **6.62** | | |  |  |
| Cigar use [PM] | **8.52** | | | **4.69** | **15.48** | | |  |  |
| Electronic vapor product use [PM]  Electronic vapor product use [LT]  Electronic vapor use at school [PM] | **2.00**  **2.02**  **2.85** | | | **1.58**  **1.60**  **2.07** | **2.57**  **2.55**  **3.93** | | |  |  |
| ***Risky Sexual behavior*** |  | | |  |  | | |  |  |
| Multiple sex partners | **2.25** | | | **1.49** | **3.41** | | |  |  |
| Having sex while drunk | **2.24** | | | **1.33** | **4.75** | | |  |  |
| ***Risky use of digital technologies*** |  | | |  |  | | |  |  |
| Play video games or use computer not for school | **1.94** | | | **1.10** | **2.34** | | |  |  |
| Talking on cell phone while driving | **2.37** | | | **2.72** | **3.29** | | |  |  |
| Texting/e-mailing while driving | **2.32** | | | **1.54** | **3.27** | | |  |  |
| ***Suicidality*** |  | | |  |  | | |  |  |
| Attempted suicide | **2.22** | | | **1.48** | **3.33** | | |  |  |
| Considered suicide | 1.50 | | | 1.10 | 2.43 | | |  |  |
| ***Aggressive behaviors [PM]*** |  | | |  |  | | |  |  |
| Risk activity on school property [carried a weapon on school property | **1.40** | | | **1.06** | **3.15** | | |  |  |
| Physical fight | **3.21** | | | **2.47** | **4.16** | | |  |  |

| **Supplemental Table 2. Perceived health status, academics and social support stratified by gambling status [N=1,807]** | | | | | | | | | | | | | |  |
| --- | --- | --- | --- | --- | --- | --- | --- | --- | --- | --- | --- | --- | --- | --- |
|  |  | |  | | | | |  | | |  |  |  |  |
|  |  | **Reported past-year gambling vs.**  **did not report past-year gambling** | | | | | | | |  |  |  |  |  |
| **Variables** | **Statistical Test** | | | | | | | |  |  |  |  |  |  |
|  | **Crude Odds Ratios** | | | **95% Confidence Limits** | | | | | | |  |  |  |  |
| ***Perceived health status*** |  | | | |  |  | | | |  | |  |  |  |
| Dysphoria/depression | 1.03 | | | 0.89 | | | 1.33 | | | |  |  |  |  |
| Considered general health as good | 1.26 | | | 0.99 | | | 1.59 | | | |  |  |  |  |
| Slept 8 hour or more | 0.83 | | | 0.62 | | | 1.10 | | | |  |  |  |  |
| ***Academics*** |  | | | | | | | | | | | |  |  |
| Mostly A and B grades | **0.45** | | | **0.35** | | | **0.59** | | | |  |  |  |  |
| Received special education | 0.88 | | | 0.62 | | | 1.27 | | | |  |  |  |  |
| ***Social Support*** |  | | |  | | |  | | | |  |  |  |  |
| Family support | **0.60** | | | **0.45** | | | **0.80** | | | |  |  |  |  |
| Teacher support | 0.75 | | | 0.59 | | | 0.94 | | | |  |  |  |  |
|  |  | | |  | | |  | | | |  |  |  |  |
